# Measuring the combinatorial expression of solute transporters and metalloproteinases transcripts in colorectal cancer

**DOI:** 10.1186/1756-0500-2-164

**Published:** 2009-08-19

**Authors:** Caroline A Kerr, Robert Dunne, Barney M Hines, Michelle Zucker, Leah Cosgrove, Andrew Ruszkiewicz, Trevor Lockett, Richard Head

**Affiliations:** 1 CSIRO Preventative Health Flagship, CSIRO Division of Molecular and Health Technologies, CSIRO, Division of Molecular and Health Technologies, Adelaide, SA, 5000, Australia; 2 CSIRO, Division of Molecular and Health Technologies, North Ryde, NSW, 1670, Australia; 3 CSIRO, Mathematical and Information Sciences, North Ryde, NSW, 1670, Australia; 4 CSIRO Division of Livestock Industries, Queensland Biosciences Precinct, St Lucia, Qld, 4067, Australia; 5 CSIRO, Division of Molecular and Health Technologies, Adelaide, SA, 5000, Australia; 6 Department of Pathology, The University of Adelaide, Adelaide, SA, 5005, Australia

## Abstract

**Background:**

It was hypothesised that colorectal cancer (CRC) could be diagnosed in biopsies by measuring the combined expression of a small set of well known genes. Genes were chosen based on their role in either the breakdown of the extracellular matrix or with changes in cellular metabolism both of which are associated with CRC progression

**Findings:**

Gene expression data derived from quantitative real-time PCR for the solute transporter carriers (SLCs) and the invasion-mediating matrix metalloproteinases (MMPs) were examined using a Linear Descriminant Analysis (LDA). The combination of MMP-7 and SLC5A8 was found to be the most predictive of CRC.

**Conclusion:**

A combinatorial analysis technique is an effective method for both furthering our understanding on the molecular basis of some aspects of CRC, as well as for leveraging well defined cancer-related gene sets to identify cancer. In this instance, the combination of MMP-7 and SLC5A8 were optimal for identifying CRC.

## Findings

Colorectal cancer is the third-most common cancer in males and second-most common in females worldwide [1]. Its prevalence highlights a need to more deeply understand the molecular interactions that lead to its progression. Two important and well documented pathways in the progression of colorectal cancer are changes in energy source for cellular metabolism and break down of the extracellular matrix.

Healthy colonocytes use short-chain monocarboxylates, in particular butyrate, as their main source of energy [2]. The solute-linked carrier (SLC) SLC5A8, a Na^+^-coupled transporter, and monocarboxylate transporter (MCT1) SLC16A, are possibly vehicles by which short-chain monocarboxylates are transported into the colonic epithelium [3-5]. SLC5A8 and SLC16A1 have been purported to provide a mechanism for the suppression of tumour growth in colorectal and gastric cancers [3,6] and are down-regulated with tumour progression [4]. As colonocytes become cancerous there is a shift in energy source away from butyrate to glucose, resulting in increased levels of glucose in colorectal cancer cells [7] and in carcinomas [8]. Associated with this is an up-regulation of the glucose transporter SLC2A1, which has been shown in a significant proportion of aggressive human tumours [e.g. [9]]. Together, these changes are believed to facilitate tumour growth and proliferation [10].

Matrix metalloproteinases (MMPs) are a family of zinc- and calcium-dependent proteolytic enzymes that degrade macromolecules of the extracellular matrix. Members of this family, such as MMP-2, -9 and -7, have been shown to be associated with the breakdown of type IV collagen and the basement membrane. They have been implicated in tumour progression and invasion in human cancer tissues [11-13]. The proteolytic activity of some MMPs (e.g. MMP-2, -9 and -14) can be suppressed by Reversion-inducing cysteine-rich protein with kazal motifs (RECK) [14]. Decreased expression of RECK is believed to result in increased invasion, metastasis and angiogenesis [reviewed by [15]] and is associated with poor prognosis in cancer patients [16].

This paper investigates genes in combination from two previous well defined processes in colorectal cancer. The abundance of transcripts from well described candidate genes implicated in either the tumorigenic process or metabolic changes associated with carcinogenesis were examined in human colorectal cancer cell lines and human cancer and healthy colonic tissues. In particular, the expression of the nutrient transporter genes (SLC2A1, SLC16A1 and SLC5A8), genes encoding proteins involved in tissue remodelling and tumour invasion (MMP-2, -7, -9 and -12, and the MMP regulator RECK), were examined in two sets of normal human colon and colorectal tumour samples and in four human colorectal cancer cell lines. The study used a combinatorial transcript expression bioinformatic approach to leverage described information on a small gene set in order to discriminate between normal and colorectal tumour tissue and help to define interrelationships between processes known to change during carcinogenesis.

## Methods

### Sample collection

Human colon tissue was sourced from the Division of Tissue Pathology, Institute of Medical and Veterinary Science, University of Adelaide. There were two sets of normal and CRC tissues as outlined in Table 1 (for further details of these samples [see Additional file 1 Tables S1 and S2].

**Table 1:** Summary of tissue sample details^

	Site	Normal	Tumour pathology			
			Dukes A	Dukes B	Dukes C	Dukes D
Study 1	Left	4	2	1	2	-
	Right	1	2	-	2	-
Study 2	Left	6	1	4	2	-
	Right	6	1	4	-	1
	Transverse	2	-	-	1	-

^see Additional file 1 for more details.

### Total RNA extraction, cDNA synthesis and real-time PCR

The human tissue samples were obtained from resections of specimens and placed in OCT (optimal cutting temperature cryopreservation medium) [17], snap-frozen in liquid nitrogen and then stored at -86°C. After histological verification RNA was extracted by placing samples in 1 ml of Trizol^® ^Reagent (Invitrogen, Sydney, Australia), then homogenised using beads (mix of 2.5 mm glass and 0.1 – 1.0 mm diameter silicon-zirconium beads) in a MiniBeadbeater-8™ (BioSpec Products Inc., Oklahoma, USA) and extracted according to Invitrogen's instructions. Samples were then further processed using RNAeasy mini spin columns (QIAGEN, Doncaster, Australia) with contaminating DNA being removed via DNase on-column digestion as per the manufacturer's instructions. Similarly, cultured cells that were at least 70% confluent were extracted directly using the RNAeasy spin columns. The integrity of RNA samples from Study 2 and the cell lines were checked using a Bioanalyzer 2100 (Agilent Technologies) [18]. All of the RNA samples were then quantified using a NanoDrop^® ^ND-1000 Spectrophotometer. Samples were then diluted to100 ng/ul.

cDNA was synthesised using SuperScript II (Invitrogen) reverse transcriptase (Invitrogen) using 1 ug of RNA per 20 uL reaction and incubated as outlined in the manufacturer's instructions. Real-time PCR assays were conducted using off-the shelf optimised and guaranteed TaqMan^® ^Gene Expression Assays (Applied Biosystems, Foster City, California, USA), that consisted of primers and a probe for the specific genes (Table 2). Three housekeeping genes were used: the conventional reference gene 18S ribosomal RNA (18S) [e.g. [19]], as well as a ribosomal protein (large P0, a component of the 60S subunit) and HUWE1 (see Table 2). The latter two were identified as suitable using a commercial database from Gene Logic (Gaithersburg, Maryland, U.S.A.). The database contains information on 44928 probe-sets (HUG-133A and B Affymetrix arrays) derived from 462 individuals covering the classes: normal 222; adenoma 29; cancer 161; and other disease 50. The dataset was normalized using the GCRMA algorithm [20] and then probe-sets were selected that had minimal variance across all arrays and had appreciable expression levels. The aim was to use three housekeeping genes of varying abundance (18s as the highest and HUWEI as the lowest) to cover the range of target transcript differential expression. Also, the target and housekeeping gene assays were selected where possible to prime over an intron-exon boundary to avoid amplifying any contaminating genomic DNA (which could otherwise bias results). The assays were then set up in four aliquots per cDNA sample using TaqMan^® ^Universal PCR Master Mix commensurate with the manufacture's instructions, except that 5 μl reaction volumes were used. Assays consisted of 20× mix of unlabeled PCR primers and TaqMan^® ^MGB probe (FAM dye-labelled). Assays were run in 384-well plates on an Applied Biosystems PRISM^® ^7900HT real-time thermocycler and analysed using Sequence Detection System software (version 2.3) as outlined in the TaqMan^® ^Gene Expression Assays protocol.

**Table 2:** Gene and assay details.

**Gene Name and Symbol**	**Genbank accession numbers**	**TaqMan Primer/Probe ID**	**Product size** **(bp)**
Eukaryotic 18S rRNA.	X_03205.1	Hs99999901_s1	187
HECT, UBA and WWE domain containing 1 (HUWE1).	NM_031407 NM_005703 NM_017627 XM_497119	Hs00328354_m1	74
Ribosomal protein, large, P0 (60s).	NM_053275.3 NM_0011002.3	Hs99999902_m1	105
Matrix metalloproteinase 12 (MMP12).	NM_002426	Hs00159178_m1	62
Matrix metalloproteinase 2 (MMP2) (gelatinase A, 72 kDa gelatinase, 72 kDa type IV collagenase).	NM_004530	Hs00234422_m1	83
Matrix metalloproteinase 7 (MMP7).	NM_002423	Hs00159163_m1	101
Metalloproteinase 9 (MMP9)(gelatinase B, 92 kDa gelatinase, 92 kDa type IV collagenase).	NM_004994	Hs00234579_m1	54
Reversion-inducing-cysteine-rich protein with kazal motifs (RECK).	NM_021111	Hs00221638_m1	76
Solute carrier family 2 (facilitated glucose transporter) member 1: SLC2A1, alias Glut1.	NM_006516	Hs00197884_m1	70
Solute carrier family 5 (iodide transporter), member 8.	NM_145913	Hs00377618_m1	88
Solute carrier family 16, member 1 (monocarboxylic acid transporter 1).	NM_003051	Hs00161826_m1	110

### Data acquisition

Data on the expression levels of target and reference genes were obtained in the form of crossing points [21] or threshold (Ct) values. The target genes were then analysed following the delta-delta Ct value procedure [22,23] with the assumption that efficiency was 100% and amplicons doubled each cycle. Briefly, the Ct for each housekeeping gene (HK) was subtracted from each corresponding target gene such that,



The mean of the normal tissues was used to create a reference tissue value. So,



and then,



This process allowed for the data to be analysed for artefacts, real-time PCR repeatability and stability of HK expression. As three HK genes were used, the process was then repeated with each housekeeping gene and the median calculated; the ratio of the gene expression is 2^-ΔΔCt^.

The normalised ΔΔCt data sets were then combined and all subset variable selection with Linear Discriminant Analysis (LDA) was performed to ascertain the best combination of transcripts that separated tumour from normal. The error rate for the model was estimated using 'leave-one-out estimates' for cross validation [24].

Transcript expression from cultured colorectal cancer cell lines (HT29, HCT116, Caco2 and LIM1215) was then used to further test the optimal combinations using LDA. The effect of tissue sampling site (i.e. left, transverse or right colon), the type of 'normal' and Dukes stage was also analysed.

## Results

**Figure 1:**
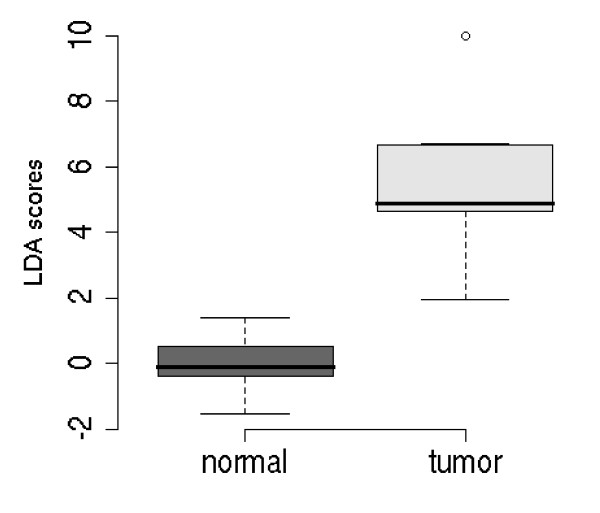
**Box plot of the optimal transcript model for separating normal (healthy) and tumour colon tissue from Study 1**. LDA scores resulted from the combined normalised gene data set reduced down to an optimal model of RECK and MMP-7.

The normalised data sets for all seven target genes for study 1, which consisted of randomly grouped tumour and normal, were analysed using LDA [see Additional file 1 for figure S1, Additional file 2 for the raw Ct values], resulting in a separation of the normal and tumour samples (the leave-one-out error estimate from the LDA is 0.25, [see Additional file 1 – Figure S2]. Using an all subsets variable selection procedure with LDA created a reduced model using only RECK and MMP-7 (see Figure 1), giving an improved leave-one-out error estimate of 0.06. Note, all the 'normal' samples clustered together regardless of their source (i.e. from a CRC or healthy patient). Therefore, there was no effect by the source of 'normal'.

**Figure 2:**
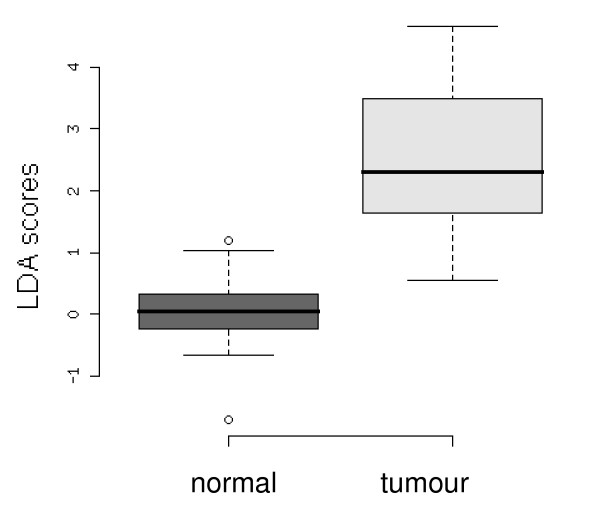
**Box plot of the optimal transcript model for separating normal (healthy) and tumour colon tissue from Study 2**. LDA scores resulted from the combined normalised gene data reduced down to an optimal model of MMP7 and SLC5A8.

For study 2, which consisted of paired data, the normalised data sets for all seven target genes were then analysed using LDA [see Additional file 1 – Figure S3 and Additional file 3 for raw Ct values], resulting in separation of the normal and tumour samples (the leave-one-out error estimate from the LDA is 0.178, [see Additional file 1 figure S4]. This separation was further increased using an all subsets variable selection procedure with LDA. The resultant reduced model, this time using only MMP7 and SLC5A8, had a leave-one-out error estimate from the LDA of 0.035 (see Figure 2).

**Figure 3:**
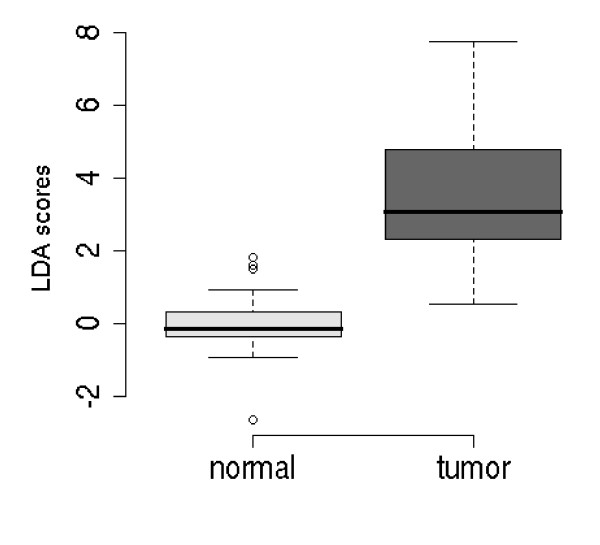
**Box plot of the optimal transcript model for separating normal (healthy) and tumour colon tissue from Studies 1 and 2**. LDA scores resulted from the combined normalised gene data set reduced down to an optimal model of MMP7 and SLC5A8.

When data from both the human tissue studies were combined and LDA applied to the four classes (Study 1 normal, Study 1 tumour, Study 2 normal, Study 2 tumour); the two sets of 'normals' could not be separated and the two sets of 'tumours' could not be separated. This indicates that it is feasible to combine the studies. The optimal transcript combination that separated tumour from normal was SLC5A8 and MMP-7 (see Figure 3) resulting in a leave-one-out error estimate of 0.128. There was no effect of sampling site (i.e., left, right or transverse colon) or Duke's stage on the expression of MMP-7 or SLC5A8.

**Figure 4:**
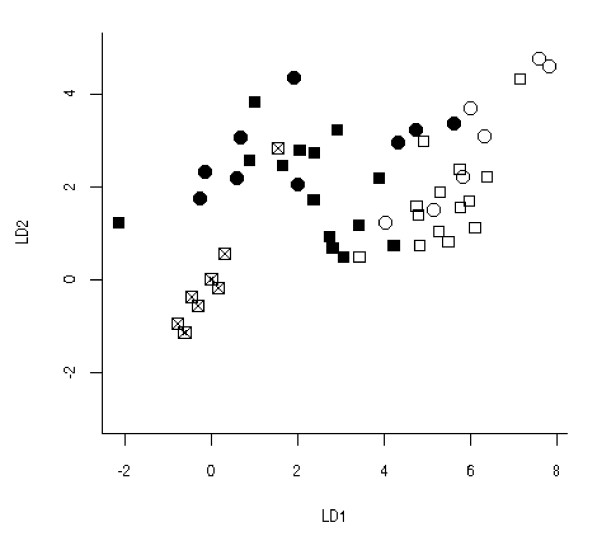
**The first two Linear Discriminants (LD1 and LD2) from Linear Discriminant Analysis (LDA) of the combined normalised gene data sets**. Reduced down to an optimal model with the transcript combination of MMP7, SLC5A8 and RECK which separated the normal (healthy) and the tumour colon tissue from Studies 1 and 2 and the cultured cell lines of ('empty circle' Study 1 normal, 'empty square' Study 2 normal, 'black circle' Study 1 tumour, 'black square' Study 2 tumour, 'black square with cross' cell lines).

Then, as an exercise to test the mathematics of this approach, the two studies plus CRC cell line data [see Additional file 1 for Figure S5 and Additional file 3 for raw Ct values] were then combined. The maximum separation between tissue types (normals, as opposed to tumour tissue plus cell lines) occurred with the transcript combination of MMP-7, RECK and SLC5A8 (see Figure 4).

## Discussion

This communication investigated expression patterns of transcripts associated with processes involved in the development of colorectal cancer. Genes examined were the solute transporters SLC2A1, SLC5A8 and SLC16A1, which are associated with changes in the cellular import of energy sources, and MMP-2, MMP-7, MMP-9 and MMP-12, which are related to the breakdown of the extracellular matrix, and the MMP negative regulator, RECK. Individual differential gene expression patterns were established for normal and cancerous tissue samples. When the data were combined, a combination of MMP-7 and SLC5A8 (and, to a lesser extent, RECK) provided the greatest separation between healthy colon tissue and colorectal cancer (tissue or cell lines). One possible interpretation of these results is that the mechanisms which act to break down the extracellular matrix and promote tumour invasion also induce MMP negative regulation. Whilst in parallel, SLC5A8 levels in tumours were reduced compared to normal tissue and cell lines, which is consistent with previous studies [4] showing an association between SLC5A8 down-regulation and tumour progression.

This study has demonstrated that it is advantageous to use a combinatorial approach to defining biomarkers of carcinogenesis processes compared to using individual candidate transcript markers. Others have used systematic approaches when analysing transcripts for cancer biomarkers (e.g. pancreatic cancer by [25]) and have shown that markers, which individually are suboptimal, can be combined to yield higher sensitivity and specificity. Even though our study uses a small patient tissue library, it demonstrates a proof-of-concept for the combinatorial approach to transcript biomarkers that now needs to be validated in larger controlled data sets [26,27]. In addition, our technique may prove useful to validate other colorectal cancer candidate transcripts, such as those defined in a recent study [28] which applied a meta-analysis or genome wide studies (e.g. microarrays) to comprehensively evaluate microarray data for biomarkers. Although using tumour-related gene expression may not be an optimal platform for colorectal cancer detection, this combinatorial approach demonstrates a method for biomarker discovery based on *a priori *hypotheses originating from other studies that may prove useful either in elucidating early biomarkers or in establishing auxiliary markers of prognosis. This approach could be applied in the clinical setting to increase the sensitivity and specificity of biomarkers by combining the analyses with other markers [29].

## Competing interests

The authors declare that they have no competing interests.

## Authors' contributions

CK designed the study and co-authored the manuscript with RD, BMH, LC, MZ, AR, TL and RH. BMH and MZ performed the molecular work. RD performed the mathematical and statistical analysis. AR provided the clinical guidance, pathology details and sourced and prepared the tissue samples. TL and RH sought the funding. All authors read and approved the final manuscript.

## Supplementary Material

Additional file 1 **Further breakdown and details of tissue and cell expression data**. Further details on the methods and results used in this study. Contains Tables S1 and S2 and Figures S1–S5. Click here for file

Additional file 2 **Table of PCR Ct values for the house keeper genes 18s, HUWE1 and RP0 and target genes MMP2, MMP12, MMp7, MMP9, RECK, SLC2A1, SLC5A8 and SLC16A1 for study1 and Cell lines (Caco2, HT29, HCT116 and LIMS1215)**. Worksheets containing raw Ct values for house keeper and target genes for study 1 (unpaired data) and cell lines. Click here for file

Additional file 3 **Table of PCR Ct values for the house keeper genes 18s, HUWE1 and RP0 and traget genes MMP2, MMP12, MMp7, MMP9, RECK, SLC2A1, SLC5A8 and SLC16A1, for Study 2**. Worksheets containing raw Ct values for house keeper and target genes for study 2 (paired data). Click here for file
