# Supplementary material for: Measuring the combinatorial expression of solute transporters and metalloproteinases transcripts in colorectal cancer
Source: BMC Res Notes. 2009 Aug 19;2:164. doi: 10.1186/1756-0500-2-164 (PMC2736974; doi:10.1186/1756-0500-2-164)
Supplement: Additional file 1 — Further breakdown and details of tissue and cell expression data. Further details on the methods and results used in this study. Contains Tables S1 and S2 and Figures S1–S5. [file 1756-0500-2-164-S1.doc]

***Additional file 1:***

***Further breakdown and details of tissue and cell expression data:***

**Additional Methods:**

Further details on the breakdown of the human tissue groups appear in Tables S1 and S2.

The cultured colorectal cancer cell lines Caco-2, HT29, and HCT 116 were used to mathematically verify analysis techniques used on human tissue sample. The cell lines were sourced and grown in accordance with American Type Culture Collection (ATCC) guidelines, with a fourth cell line (LIM 1215) grown as previously described[[1]](#footnote-2).

**Further Results:**

***Human Tissue Study 1:***

The human tissue (normal colon and tumour) normalised (to the house keeper median) gene expression results are illustrated in Figure S1; they reveal a large variation in expression levels within either group (normal or tumour) for MMP-2, MMP-9, MMP-12 and SLC2A1. The genes MMP-7, RECK, SLC5A8 and SLC16A1 were significantly differentially expressed (p = 0.02, 0.0002, 0.04, 0.04; respectively) between the two tissue groups. However, it is important to note that SLC5A8 expressions levels were low and at the limits of the assay’s detection. The normalised data sets for all seven target genes were then analysed using LDA (see Figure S2), resulting in a separation of the normal and tumour samples (the leave-one-out error estimate from the LDA is 0.25). Using an all subsets variable selection procedure with LDA created a reduced model using only RECK and MMP-7, giving an improved leave-one-out error estimate of 0.06. However, even though RECK and MMP-7 were the optimal transcript combinations, any combination of the transcripts that were significantly differentially expressed on an individual basis gave a similar combined separation between tumour and normal.

***Human Tissue Study 2:***

The human tissue (normal colon and tumour) gene expression results for study 2 (paired and normalised to the HK median) are illustrated in Figure S3. Similar to study 1, a large variation in expression levels was demonstrated within the two groups (normal and tumour) for MMP-2, MMP-9, RECK and SLC2A1 (i.e., p>0.05). Of the transcripts, MMP-7, MMP-12, SLC5A8 and SLC16A1 were differentially expressed (p = 0.0001, 0.012, 0.001 and 0.003; respectively) between the two tissue groups. The normalised data sets for all seven target genes were then analysed using LDA (see Figure S4), resulting in separation of the normal and tumour samples (the leave-one-out error estimate from the LDA is 0.178). This separation was further increased using an all subsets variable selection procedure with LDA. The resultant reduced model, this time using only MMP7 and SLC5A8, had a leave-one-out error estimate from the LDA of 0.035. Again, a combination of either of the individually differentially expressed gave a similar combined separation between tumour and normal as MMP-7 and SLC5A8. Therefore, the results are consistent for each study for differential expression of transcript combinations of MMP-7, SLC5A8 and SLC16A1.

***Cell line studies and their combination with human tissue studies:***

There were distinct differential patterns of normalised expression of SLC2A1, SLC5A8, MMP-2, MMP-7, MMP-9 and RECK across the four cell lines (see Figure S5). These data were then combined with the normalised expression data for all the transcripts from the two colon tissue sample studies. The maximum separation between tissue types (normals, as opposed to tumour tissue plus cell lines) occurred with the transcript combination of MMP-7, RECK and SLC5A8.

**Table S1: Pathology details of patient samples of normal (healthy colon or non-tumour involved colon from colorectal cancer patients) or tumour material from Study 1.**

| **Number** | **Patient details** | **Tissue Pathology** | **Tissue sampling site** |
| --- | --- | --- | --- |
| 1 | CRC (T3, Dukes B) | Normal | Left colon |
| 2 | CRC (T2, Duke A) | Normal | Left colon, |
| 3 | Healthy | Normal | Unknown |
| 4 | Healthy | Normal | Unknown |
| 5 | CRC (T3, Duke C, mucinous) | Normal | Left colon |
| 6 | CRC (T1, Duke A) | Normal | Right colon |
| 7 | CRC (T3,Duke B) | Normal | Left colon |
|  |  |  |  |
| 8 | CRC | Tumour T3, Dukes C | Left colon |
| 9 | CRC | T2, Dukes A, mucinous | Left colon |
| 10 | CRC | T3, Dukes C, mucinous | Right colon |
| 11 | CRC | T2, Dukes A, mucinous | Right colon |
| 12 | CRC | T3, Dukes B, | Left colon |
| 13 | CRC | Adenoma | Right colon |
| 14 | CRC | T1, Dukes A | Left colon |
| 15 | CRC | T3, Dukes C | Left colon |
| 16 | CRC | T3, Dukes C, mucinous | Right colon |
|  |  |  |  |

**Table S2: Human tissue samples tumour pathology from Study 2^.**

| **ID** | **G** | **Age** | **Site** | **Size**  **(mm)** | **Histo type** | **Grade** | **I.M.** | **Depth invasion** | **T** | **TILs** | **Crohn's like** | **Duke** | **Pos Nodes** | **Apical node** | **Radial margin** | **Mural vascular invasion** | **Extramural vascular invasion** | **Neural** | **MLH1** | **MSH2** | **MSH6** | **PMS2** | **MSI** |
| --- | --- | --- | --- | --- | --- | --- | --- | --- | --- | --- | --- | --- | --- | --- | --- | --- | --- | --- | --- | --- | --- | --- | --- |
|  |  |  |  |  |  |  |  |  |  |  |  |  |  |  |  |  |  |  |  |  |  |  |  |
| 1 | F | 83 | Caecum | 83 | Signet ring cell | 3 | No | Beyond MP | 3 | No | No | B | 0 | N | N/A | N | N | N | - | + | + | - | + |
| 2 | M | 72 | Recto-  sigmoid | 35 | Adenomuc | 2 | No | Beyond MP |  | No | Yes | B | 0 | N | N/A | N | N | N | + | + | + | + | - |
| 3 | F | 55 | Rectum | 50 | Adenomuc | 2 | No | Beyond MP | 3 | No | No | B | 0 | N | Free | Y | Y | N | + | + | + | + | - |
| 4 | M | 69 | Rectum | 15 | Adenomuc | 2 | No | into submucosa |  | No | No | A | 1 | N | Free | Y | N | N | + | + | + | + | - |
| 5 | F | 66 | Transverse | 12 | Adenoca | 2 | No | into submucosa |  | No | No | A | 0 | N | NA | N | N | N | + | + | + | + | - |
| 6 | F | 72 | Rectum | 30 | Adenoca | 2 | No | Beyond MP | 3 | No | No | D | 3 | N | Free | Y | Y | N | + | + | + | + | - |
| 7 | F | 77 | Ascending | 50 | Adenocamuc Signet | 3 | No | Beyond MP | 3 | Yes | Yes | B | 0 | N | N/A | Y | N | N | - | + | + | - | + |
| 8 | F | 83 | Ascending | 28 | Adenoca | 2 | No | Beyond MP | 3 | No | No | B | 0 | N | N/A | Y | Y | N | + | + | + | + | - |
| 9 | F | 43 | Rectum | 30 | Adenoca | 2 | No | Beyond MP | 3 | No | Yes | B | 0 | N | Free | Y | N | N | + | + | + | + | - |
| 10 | M | 77 | Ascending | 40 | Adenoca | 2 | Yes | Beyond MP | 3 | No | No | C | 12 | P | + | Y | Y | N | + | + | + | + | - |
| 11 | M | 66 | Rectum | 45 | Adenoca | 2 | No | Beyond MP | 3 | No | No | B | 0 | N | Free | Y | Y | N | + | + | + | + | - |
| 12 | M | 86 | Ascending | 40 | Adenoca | 2 | No | Beyond MP | 3 | No | No | B | 0 | N | N/A | Y | Y | N | + | + | + | + | - |
| 13 | F | 64 | Transverse | 100 | Adenoca | 2 | Yes | Serosa breached | 4 | N | N | C | 14 | P | N/A | Y | Y | N | - | + | + | - | + |
| 14 | M | 71 | Caecum | 35 | Adenocamuc | 2 | No | Beyond MP | 3 | N | N | C | 2 | N | N/A | N | N | N | + | + | + | + | - |

**^G = gender, S = surgery (P-palliative or C-curative), I.M. = infiltrating margin, G = Grade (2 - moderately/well differentiated, 3 – poorly differentiated), Depth invasion = beyond MP (beyond muscularis propria), T = T stage, TILS = tumour infiltrating lymphocytes, free of proximal and distal, Crohn’s = Crohn’s like aggregates and inflammatory reaction; Histo type: Adenoca = Adenocarcinoma, Adenomuc – Adenocarcinoma with mucinous component, Mucinous Carcinoma, Adenocarcinoma with Mucinous component and signet ring cells, Pos Nodes= position Nodes, MLH1, MSH2, MSH6, PMS2 (- abnormal expression, + normal expression).**


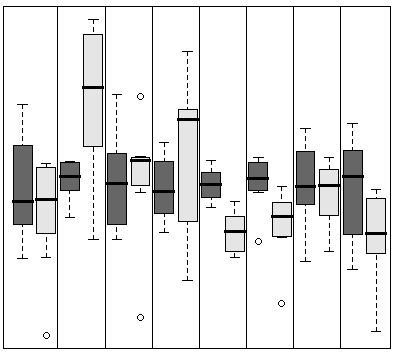


**MMP-2 MMP-7 MMP-9 MMP-12 RECK SLC5A8 SLC2A1 SLC16A1**

**Figure S1: Box plots of normalised expression of the target genes MMP2, MMP-7, MMP-9, MMP-12, RECK, SLC5A8, SLC2A1 and SLC16A1, across seven normal (healthy) colon (◼) and nine colorectal cancer tissue samples (◼) from Study 1.**


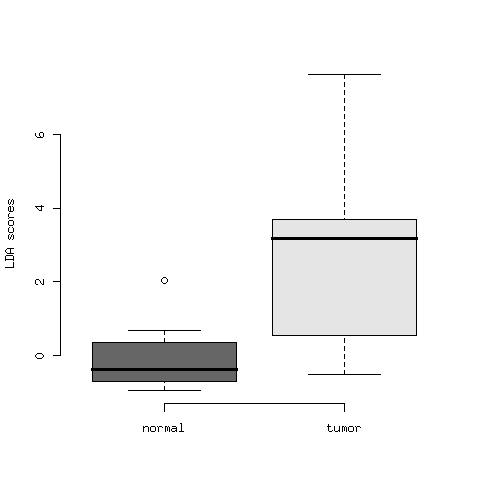


**Figure S2: Box plot of the data in Figure 1 as LDA scores for the normal (healthy) and the tumour-affected colon tissue for the combined normalised target gene data set from Study 1 (consisting of SLC5A8, SLC2A1, MMP-2, MMP-7, MMP-9, MMP-12 and RECK).**

**Figure S3: Normalised expression of the target genes. A: MMP-2, B: MMP-7, C: MMP-9, D: MMP-12, E: RECK, F: SLC5A8, G: SLC2A1 and H: SLC16A1, across 14 colorectal cancer tissue and paired ‘normal’ colon samples from each patient (vertical line) in Study 2.**

**
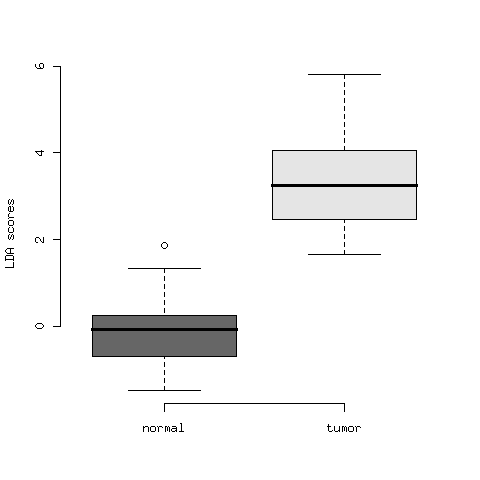
**

**Figure S4: Box plot of the LDA scores for the normal (healthy) and the tumour-affected colon tissue for the combined normalised target gene data set from Study 2 (consisting of SLC5A8, SLC2A1, SLC16A1, MMP-2, MMP-7, MMP-9, MMP-12 and RECK).**

**Figure S5: Normalised expression of the target genes (n=3 replicates), A: MMP-2, B: MMP-7, C: MMP-9, D: MMP-12, E: RECK, F: SLC5A8, G: SLC2A1 and H: SLC16A1 across the cell lines HT29, HCT116, Caco2 and LIM1215.**

1. Whitehead RH, Young GP, Bhathal PS: [**A new hormone responsive Colon-Carcinoma Cell-Line (LIM1215) capable of differentiation**](http://apps.isiknowledge.com:80/WoS/CIW.cgi?SID=3FiHDK5g34E3loCpDDe&Func=Abstract&doc=13/10) ***in vitro*.** *Gastroenterol* 1985, **88:**1630-1630. [↑](#footnote-ref-2)
